# Colorectal Cancer Screening in Average Risk Populations: Evidence Summary

**DOI:** 10.1155/2016/2878149

**Published:** 2016-08-14

**Authors:** Jill Tinmouth, Emily T. Vella, Nancy N. Baxter, Catherine Dubé, Michael Gould, Amanda Hey, Nofisat Ismaila, Bronwen R. McCurdy, Lawrence Paszat

**Affiliations:** ^1^Prevention and Cancer Control, Cancer Care Ontario, Toronto, ON, Canada; ^2^Department of Medicine, Sunnybrook Health Sciences Centre, Toronto, ON, Canada; ^3^Institute for Clinical Evaluative Sciences, Toronto, ON, Canada; ^4^Institute of Health Policy, Management and Evaluation, Dalla Lana School of Public Health, University of Toronto, Toronto, ON, Canada; ^5^Department of Medicine, University of Toronto, Toronto, ON, Canada; ^6^Program in Evidence-Based Care, Cancer Care Ontario, Hamilton, ON, Canada; ^7^Department of Surgery, St. Michael's Hospital, Toronto, ON, Canada; ^8^Li Ka Shing Knowledge Institute, St. Michael's Hospital, Toronto, ON, Canada; ^9^Department of Medicine, Division of Gastroenterology, University of Ottawa, The Ottawa Hospital, Ottawa, ON, Canada; ^10^William Osler Health Centre, Etobicoke, ON, Canada; ^11^Vaughan Endoscopy Clinic, Vaughan, ON, Canada; ^12^Northeast Cancer Centre Health Sciences North/Horizon Santé-Nord, Sudbury Outpatient Centre, Sudbury, ON, Canada; ^13^American Society of Clinical Oncology, Alexandria, VA, USA

## Abstract

*Introduction*. The objectives of this systematic review were to evaluate the evidence for different CRC screening tests and to determine the most appropriate ages of initiation and cessation for CRC screening and the most appropriate screening intervals for selected CRC screening tests in people at average risk for CRC.* Methods*. Electronic databases were searched for studies that addressed the research objectives. Meta-analyses were conducted with clinically homogenous trials. A working group reviewed the evidence to develop conclusions.* Results*. Thirty RCTs and 29 observational studies were included. Flexible sigmoidoscopy (FS) prevented CRC and led to the largest reduction in CRC mortality with a smaller but significant reduction in CRC mortality with the use of guaiac fecal occult blood tests (gFOBTs). There was insufficient or low quality evidence to support the use of other screening tests, including colonoscopy, as well as changing the ages of initiation and cessation for CRC screening with gFOBTs in Ontario. Either annual or biennial screening using gFOBT reduces CRC-related mortality.* Conclusion*. The evidentiary base supports the use of FS or FOBT (either annual or biennial) to screen patients at average risk for CRC. This work will guide the development of the provincial CRC screening program.

## 1. Introduction

Canada has one of the highest rates of colorectal cancer (CRC) in the world, with an estimated 25,100 cases in 2015 [1, 2]. CRC is also one of the leading causes of cancer-related death for men and women combined in Canada, with an estimated 9300 deaths in Canada in 2015. However, if CRC is found in its early stages, there is a 90% chance that it can be cured [1]. Cancers detected through screening tend to be earlier stage compared with cancers detected outside of screening [3–6]. In 2008, Ontario launched its CRC screening program, which offers screening to Ontarians aged 50 to 74. People at average risk are offered the guaiac fecal occult blood test (gFOBT) once every two years, while people at increased risk, defined as having one or more first-degree relatives with CRC, are offered colonoscopy. In 2013, approximately 58.5% of Ontarians were up-to-date with CRC screening tests [7].

In light of emerging evidence, the provincial CRC screening program is seeking guidance for CRC screening of average risk individuals in Ontario. Cancer Care Ontario's Prevention and Cancer Control portfolio and the Program in Evidence-Based Care (PEBC) developed this evidentiary base to help inform the CRC screening program in Ontario, ColonCancerCheck.

The evidence supporting primary screening tests for CRC, ages of initiation and cessation for CRC screening, and screening intervals for selected CRC screening tests in people at average risk for CRC was systematically reviewed to develop this evidentiary base. Below, the methods and key findings of the systematic review are summarized. The full evidentiary base is available online [8].

## 2. Systematic Review Objectives

The purpose of this systematic review is to evaluate the existing evidence concerning screening of adults at average risk for CRC in the context of an organized, population-based screening program. The main objectives are to identify the following:

- The benefits and harms of screening in this population.
- The optimal primary CRC screening test(s) for this population.
- The appropriate ages of initiation and cessation for screening in this population.
- The intervals at which people at average risk should be recalled for CRC screening.

## 3. Target Population

The target population includes primary care providers, endoscopists, policy-makers, and program planners in Ontario.

## 4. Research Questions

### 4.1. Primary Research Question

Primary Research Question is as follows:

1. How do different screening tests, individually or in combination, perform in average risk people in preventing CRC-related mortality or all-cause mortality or in decreasing the incidence of CRC? Secondary outcomes include the detection of cancer or its precursors, screening participation rate, adverse effects of tests, and test characteristics, such as sensitivity, specificity, positive predictive value, negative predictive value, and proportion of false-positives or of false-negatives.

### 4.2. Secondary Research Questions

Secondary Research Questions are as follows:

1. What are the appropriate ages of initiation and cessation for screening in people at average risk for CRC? Is there a relationship between age and the effectiveness of CRC screening?
2. What are the appropriate intervals between CRC screening tests (by test)? Is there a relationship between screening intervals and the effectiveness and risks of screening?

## 5. Methods

The authors of this evidentiary base (working group) consisted of one primary care physician, one colorectal surgeon, one expert in public health screening, one policy analyst from the Ontario CRC screening program, two methodologists, and three gastroenterologists. The PEBC, a provincial program of Cancer Care Ontario, is supported by the Ontario Ministry of Health and Long-Term Care. All work produced by the PEBC and any associated programs is editorially independent from the ministry.

A two-stage method was used. It is summarized here and described in more detail as follows:

1. Search and evaluation of existing systematic reviews: if existing systematic reviews were identified that addressed the research questions and were of reasonable quality, then they were included as a part of the evidentiary base.
2. Original systematic review of the primary literature: this review focused on areas not covered by existing and accepted reviews.

### 5.1. Literature Search Strategy

A systematic search was conducted in OVID MEDLINE (2006 to September 3, 2014), EMBASE (2006 to September 3, 2014), the Cochrane Library (Issue: 2–4, October 2013), and the American Society of Clinical Oncology (ASCO) conference proceedings (2009 to 2013). Details of the literature search strategy can be found online [8].

#### 5.1.1. Study Selection Criteria and Protocol

Systematic reviews were included if

- they addressed at least one of the research questions,
- they evaluated randomized or nonrandomized control trials of asymptomatic average risk subjects undergoing CRC screening,
- the literature search strategy for the existing systematic review was reproducible (i.e., reported) and appropriate,
- the existing systematic review reported the sources searched, as well as the dates that were searched.

Identified systematic reviews were assessed using the Assessing of Methodological Quality of Systematic Reviews (AMSTAR) tool [82]. In cases where multiple systematic reviews were identified for a particular outcome, only evidence from the most recent systematic review with the highest quality was used in the evidence base. The literature was searched for new primary studies published after the end search date of included systematic reviews. Individual study quality from the studies included in the systematic reviews as well as any new primary studies was assessed in order to complete the Grading of Recommendations, Assessment, Development and Evaluations (GRADE) tables for risk of bias [9].

If no existing systematic review was identified for a given test or question, or if identified reviews were incomplete, a systematic review of the primary literature was performed. Articles in reference lists from included studies were also searched. The scope of the primary literature review was tailored to address the gaps in the incorporated existing systematic reviews (e.g., subject areas covered and time frames covered). The criteria for the primary literature are described as follows.

*Inclusion Criteria.* Inclusion criteria are as follows:

(1) Randomized controlled trials (RCTs) (primary research question and secondary research questions 1 and 2) that could be identified directly from the search or from reference sections of systematic reviews.
(2) Cohort/case-control studies, minimum study size *n* = 30 (secondary research questions 1 and 2).
(3) Evidence from nonrandomized prospective comparative studies with historical or contemporaneous controls, with the consensus of the working group, when there were gaps in available evidence from RCTs.
(4) Studies preferred with asymptomatic average risk subjects and population-based studies that did not oversample adults with symptoms of CRC or a family history of CRC which were also considered acceptable.
(5) For conference abstracts: RCTs (all questions).
(6) The following screening tests considered for inclusion:
  (i) fecal-based tests including gFOBT, fecal immunochemical test (FIT), stool DNA panel (stool DNA), and fecal M2-PK,
  (ii) blood tests (Cologic®, ColonSentry®, mSEPT9, metabolomics, and hydroxylated polyunsaturated long chain fatty acids),
  (iii) endoscopic tests including flexible sigmoidoscopy (FS), colonoscopy, and capsule colonoscopy,
  (iv) radiological tests including double-contrast barium enema (DCBE) and computed tomography colonography (CT colonography).

*Exclusion Criteria.* Exclusion criteria are as follows:

1. Letters, comments, or editorials.
2. Studies that included a population enriched with subjects with symptoms of CRC or a family history of CRC.
3. Nonsystematic reviews.
4. Non-English-language publications.

One of the two reviewers (NI and EV) independently reviewed the titles and abstracts resulting from the search. For items that warranted full-text review, NI or EV reviewed each item independently. However, in uncertain cases, a second reviewer (JT) was asked to review them.

#### 5.1.2. Data Extraction and Assessment of Study Quality and Potential for Bias

Data from the included studies were independently extracted by NI and EV. If there was more than one publication for the same study, only the most updated or recent versions of the data were reported in the result. All extracted data and information were audited by an independent auditor.

Important quality features, such as randomization details, sample size and power, intention-to-screen (ITS) analysis, length of follow-up, and funding, for each RCT, were extracted. The quality of observational studies was assessed using a modified Newcastle-Ottawa Scale [83]. The quality of diagnostic studies was assessed using a modified Quality Assessment of Diagnostic Accuracy Studies (QUADAS) tool [84]. The GRADE method for assessing the quality of aggregate evidence was used for each comparison using the GRADEpro Guideline Development Tool [9, 85]. The working group used the GRADE system for ranking outcomes and scored each outcome from the evidence review on a scale from 1 to 9. Outcomes with a score from 1 to 3 were considered of limited importance, from 4 to 6 important, and from 7 to 9 critical in the development of recommendations for the CRC screening program. Only outcomes that were considered critical or important were included in the GRADE evidence tables.

#### 5.1.3. Synthesizing the Evidence

When clinically homogenous results from two or more trials were available, a meta-analysis was conducted using review manager software (RevMan 5.3) provided by the Cochrane Collaboration [86]. For all outcomes, the dichotomous model with random effects was used. The number of person-years, rather than the total number of subjects, was used, if available. The number of person-years takes into account the fact that different people in the study may have been followed up for different lengths of time.

In order to have comparable control rates across all gFOBT and FS trials, the control rates for the no screening groups in the gFOBT and FS trials were combined and calculated from the total number of cases across all gFOBT and FS trials over the total number of person-years across all gFOBT and FS trials.

Statistical heterogeneity was calculated using *χ* ^2^ test for heterogeneity and *I* ^2^ percentage. A probability level for *χ* ^2^ statistic less than or equal to 10% (*p* ≤ 0.10) and/or an *I* ^2^ greater than 50% was considered indicative of statistical heterogeneity.

#### 5.1.4. Process for Developing Conclusions

The working group members met in person on four occasions to develop evidence-based conclusions through consensus. For each comparison (e.g., gFOBT versus no screening) the working group assessed the quality of the body of evidence for each outcome using the GRADE process [9]. Five factors were assessed for each outcome in each comparison: the risk of bias, inconsistency, indirectness, imprecision, and publication bias. Observational studies were initially graded as low quality and RCTs as high quality; the quality of the evidence was downgraded when serious threats were identified to one or more factors. At the in person meetings, the working group discussed each comparison and agreed on the overall certainty of the evidence across outcomes (Table 1), whether the desirable anticipated effects were large, whether the undesirable anticipated effects were small, and whether the desirable effects were large relative to the undesirable effects. For each comparison, conclusions were developed that reflected these working group discussions.

## 6. Results

### 6.1. Literature Search Results

A total of 7538 studies were identified and 378 were selected for full-text review. Of those, 48 met the predefined eligibility criteria for this systematic review. An additional 27 articles were found from the reference lists. After our literature search, we became aware of and included an updated publication for one of the FS screening RCTs that had already been identified [21]. A total of 76 articles were included of which eight were systematic reviews [10, 29, 30, 59, 68, 87–89], 39 [6, 11–28, 60–66, 69–81] were from 30 RCTs, 19 were prospective studies [31–43, 52, 53, 55–57, 67], five were retrospective studies [44–48], and five were case-control studies [49–51, 54, 58]. Evidence from five of the eight systematic reviews was included either because the reviews were the most recent systematic review with the highest quality evidence for a particular outcome or because they included an outcome of interest not covered by other high-quality reviews [10, 29, 30, 59, 68]. After the search process and quality assessment, a total of 73 articles were included in this systematic review. The search flow diagram, the characteristics and quality of the included systematic reviews and studies, and the meta-analyses can be found online or in Supplementary Figures  1 to  19 (see Supplementary Material available online at http://dx.doi.org/10.1155/2016/2878149) [8].  Table 2 provides a summary of the number and type of studies used for each comparison.

## 7. Interpretation and Conclusions

The following are the conclusions developed by the working group based on the review of the evidence and meta-analyses. When discussing the effects of various screening tests, the reported outcomes vary by test. There was strong agreement among the members of the working group that CRC-related mortality and complications from screening tests were critical outcomes for recommendation development. All-cause mortality, CRC incidence, participation rate, and diagnostic outcomes were considered important outcomes of interest.

### 7.1. Fecal Tests for Occult Blood

There was strong evidence to support the use of fecal tests for occult blood to screen people at average risk for CRC.

#### 7.1.1. Guaiac Fecal Occult Blood Test (gFOBT) versus No Screening

- The overall certainty of the evidence was high, suggesting a definite reduction in CRC-related mortality (Table 3 and Supplementary Figures  1 to  3). The magnitude of the effect was small (relative risk [RR], 0.87; 95% confidence interval [CI], 0.82 to 0.92). The magnitude of benefit was comparable to the disease-specific reduction in mortality from mammography for breast cancer screening (RR, 0.79; 95% CI, 0.68 to 0.90) [90] but was less than that from the human papillomavirus (HPV) test for cervical cancer screening (hazard ratio [HR], 0.52; 95% CI, 0.33 to 0.83) [91]. The anticipated harms associated with gFOBT (including follow-up colonoscopy for people with positive tests) are small and outweighed by the benefits.

#### 7.1.2. Fecal Immunochemical Test (FIT) versus gFOBT

- The overall certainty of the evidence was moderate (Table 4 and Supplementary Figures  7 to  10). The magnitude of the desirable anticipated effects was at least equivalent to gFOBT, and it is likely that the desirable effects of FIT are greater than for gFOBT. The anticipated undesirable effects associated with FIT (including follow-up colonoscopy for people with positive tests) are small and outweighed by the benefits.
- While there were well-designed randomized controlled trials (RCTs) comparing FIT with gFOBT, the outcomes of these trials (participation and detection rates) were considered to be less important than CRC-related mortality. However, it was anticipated that the reduction in CRC-related mortality and the complications resulting from screening with FIT would be at least equivalent to those observed from screening with gFOBT. FIT's greater sensitivity for detection of CRC and advanced adenomas compared with gFOBT suggests that the reduction in CRC incidence with FIT could be greater than with gFOBT; however, the magnitude and significance of any additional benefit of FIT over gFOBT are unknown. It is important to highlight that the FIT positivity threshold selected would be an important determinant of the magnitude of the benefits and harms of FIT relative to gFOBT.

### 7.2. Lower Bowel Endoscopy

There was strong evidence to support the use of flexible sigmoidoscopy (FS) to screen people at average risk for CRC. There was no direct evidence to support the use of colonoscopy to screen people at average risk for CRC, but evidence from FS informed the assessment of the benefits and harms of colonoscopy in screening people at average risk for CRC.

#### 7.2.1. FS versus No Screening

- The overall certainty of the evidence was high, suggesting that FS has a definite effect on CRC-related mortality and incidence when compared with no screening (Table 5 and Supplementary Figures  4 to  6). The magnitude of the effect on CRC mortality was modest (RR, 0.72; 95% CI, 0.65 to 0.80); it exceeds the anticipated disease-specific reduction in mortality from gFOBT for CRC screening (RR, 0.87; 95% CI, 0.82 to 0.92) and is similar to the effects of mammography on breast cancer mortality (RR, 0.79; 95% CI, 0.68 to 0.90) [90] and of the HPV test on cervical cancer mortality (HR, 0.52; 95% CI, 0.33 to 0.83) [91]. The effect on survival with FS was also comparable to the benefit achieved with the current standard of care for patients with completely resected stage III CRC (5-fluorouracil/leucovorin plus oxaliplatin [FOLFOX or FLOX] versus 5-fluorouracil/leucovorin alone, HR for overall survival at six years, 0.80; 95% CI, 0.65 to 0.97) [92]. The anticipated harms associated with FS (including follow-up colonoscopy for people with positive tests) were small and outweighed by the benefits.

#### 7.2.2. Colonoscopy versus No Screening

- The overall certainty of direct evidence supporting the use of colonoscopy to screen people at average risk for CRC was very low when compared with no screening (Table 6). The desirable and undesirable anticipated effects were uncertain.
- It is anticipated that the benefit of screening with colonoscopy would be at least equivalent to that observed for screening with FS; however, the magnitude of additional benefit over FS, if any, is unknown. The magnitude of additional undesirable effects of colonoscopy relative to FS is also unknown.

### 7.3. Fecal Tests for Occult Blood versus Lower Bowel Endoscopy

There was insufficient evidence to determine how fecal tests for occult blood perform compared with lower bowel endoscopy to screen people at average risk for CRC (Supplementary Tables  1 to  5 and Supplementary Figures  11 to  19).

- The studies that compared one-time fecal tests for occult blood to lower bowel endoscopy were heterogeneous, with few comparisons where data could be pooled. However, in general, the evidence suggested that participation was higher and detection rate was lower with fecal-based tests compared with endoscopic tests.
- The overall certainty of the evidence was low. CRC-related mortality was not evaluated and the design of the studies favoured endoscopic tests because the comparison was to one-time fecal-based testing (rather than repeated testing over time, which is how these tests are used in usual practice). There was significant heterogeneity in participation. The undesirable anticipated effects of endoscopy (including follow-up endoscopy for people with positive fecal tests) are probably small. It is uncertain whether the desirable effects are large relative to the undesirable effects.

### 7.4. Radiological Tests

#### 7.4.1. Computed Tomography Colonography versus Colonoscopy

There was insufficient evidence to determine how computed tomography colonography performs compared with colonoscopy to screen people at average risk for CRC (results not shown; see [8]).

- The overall certainty of the evidence was low. The desirable and undesirable anticipated effects were uncertain.

#### 7.4.2. Capsule Colonoscopy versus Colonoscopy

There was insufficient evidence to determine how capsule colonoscopy performs compared with colonoscopy to screen people at average risk for CRC (results not shown; see [8]).

- The overall certainty of the evidence was very low. The desirable and undesirable anticipated effects were uncertain.

#### 7.4.3. Double-Contrast Barium Enema (DCBE)

There was no evidence to support the use of DCBE to screen people at average risk for CRC.

- Since 2006, there has been no new published evidence on this topic. Most recent CRC guidelines except for a 2008 guideline by the American Cancer Society, the US Multi-Society Task Force on Colorectal Cancer, and the American College of Radiology [93] have not endorsed the use of DCBE for screening [94–98].

### 7.5. DNA Tests

#### 7.5.1. Stool DNA versus Fecal Occult Blood Tests (gFOBT or FIT)

There was insufficient evidence to determine how stool DNA performs compared with gFOBT or FIT to screen people at average risk for CRC (results not shown; see [8]).

- The overall certainty of the evidence was very low. The desirable and undesirable anticipated effects were uncertain.

#### 7.5.2. Other DNA Tests

There was insufficient evidence to support the use of mSEPT9 to screen people at average risk for CRC (results not shown; see [8]).

- The overall certainty of the evidence was very low. The desirable and undesirable anticipated effects were uncertain.

### 7.6. Metabolomic Tests

#### 7.6.1. Fecal M2-PK

There was insufficient evidence to support the use of fecal M2-PK to screen people at average risk for CRC (results not shown; see [8]).

- The overall certainty of the evidence was very low. The desirable and undesirable anticipated effects were uncertain.

#### 7.6.2. Other Metabolomic Tests

There was no evidence to support the use of other metabolomic tests (e.g., low levels of hydroxylated polyunsaturated long chain fatty acids [Cologic]) to screen people at average risk for CRC.

### 7.7. Age of Initiation/Cessation

#### 7.7.1. Age of Initiation/Cessation with gFOBT

Currently, the Ontario CRC screening program recommends that average risk individuals initiate screening with gFOBT beginning at 50 years of age and ending at age 74. There was insufficient evidence to support changing the ages of initiation and cessation for CRC screening with gFOBT in Ontario (results not shown; see [8]).

- The overall certainty of the evidence was very low. There was insufficient evidence to demonstrate differences in reduction of CRC mortality using gFOBT across age groups. The desirable and undesirable anticipated effects across age groups were uncertain.

#### 7.7.2. Age of Initiation/Cessation with FS

There was insufficient evidence to recommend ages of initiation or cessation when screening with FS in people at average risk for CRC (results not shown; see [8]).

- The overall certainty of the evidence was very low. There was insufficient evidence to demonstrate differences in reduction of CRC mortality or incidence using FS across age groups. The desirable and undesirable anticipated effects across age groups were uncertain.
- Of the four large FS RCTs, three examined “once in a lifetime” FS between the ages of 55 and 64, while the fourth RCT examined baseline FS between the ages of 55 and 74 with a second FS after three or five years.

#### 7.7.3. Age of Initiation/Cessation with Colonoscopy

There was insufficient evidence to recommend an age of initiation or cessation to screen with colonoscopy in people at average risk for CRC (results not shown; see [8]).

- The overall certainty of the evidence was very low. There was insufficient evidence to demonstrate differences in CRC detection using colonoscopy across age groups. The desirable and undesirable anticipated effects across age groups were uncertain.
- Currently, the Ontario CRC screening program does not recommend colonoscopy to screen persons at average risk for CRC. The program does recommend colonoscopy in people at increased risk (one or more first-degree relatives with CRC) starting at 50 years of age or 10 years younger than the age at which the relative was diagnosed, whichever occurred first.

#### 7.7.4. Age of Initiation/Cessation with FIT

There were no studies that met our inclusion criteria for age of initiation/cessation for FIT.

### 7.8. Screening Intervals

#### 7.8.1. gFOBT Intervals

There was evidence to suggest that either annual or biennial screening using gFOBT in people at average risk for CRC reduces CRC-related mortality (results not shown; see [8]).

- The overall certainty of the evidence was moderate. The desirable anticipated effects on CRC mortality were small and similar for annual or biennial screening. The undesirable anticipated effects were not reported for each interval group. Anticipated harms associated with gFOBT (including follow-up colonoscopy for people with positive tests) were small for biennial screening and were likely to be greater for annual screening. In addition, annual screening is anticipated to increase burden to the participant.

#### 7.8.2. FIT Intervals

There was insufficient evidence to recommend an interval to screen people at average risk for CRC using FIT (results not shown; see [8]).

- The overall certainty of the evidence was very low. The desirable and undesirable anticipated effects were uncertain.

#### 7.8.3. FS and Colonoscopy Intervals

There were no studies that met our inclusion criteria for screening intervals for FS or colonoscopy.

## 8. Summary and Next Steps

This evidentiary base summarizes the known clinical effectiveness and safety of CRC screening tests. Concurrently, the Canadian Task Force on Preventive Health Care (CTFPH) [99] and the US Preventive Services Task Force (USPSTF) have published guidelines on CRC screening [100]. The audience for the 3 reviews differed slightly: the current review seeks to provide guidance to the CRC screening program in light of emerging evidence in CRC screening while the CTFPH and the USPSTF specify “primary care physicians” and “primary care clinicians and patients” as their target audiences, respectively. All 3 reviews support the use of fecal testing (gFOBT or FIT) and flexible sigmoidoscopy but treat colonoscopy slightly differently. The CTFPH recommends against colonoscopy while the USPSTF endorses it as one of a range of options. In the current review, we grade the evidence only (recommendations for Ontario's CRC screening program are released separately; see below). Our interpretation of the evidence acknowledges that the strong evidence in favour of flexible sigmoidoscopy does inform the assessment of colonoscopy. However, this evidence is both indirect and incomplete (magnitude of additional benefit from colonoscopy and of additional harms is unknown). In order to have complete understanding of the benefits and harms of colonoscopy, we await the results of ongoing randomized controlled trials in colonoscopy for average risk screening, anticipated in the 2020s.

The evidence from the current review is central to the ongoing development of Ontario's CRC screening program. However, this evidentiary base is necessary but not sufficient to guide program development as other context-specific criteria such as cost-effectiveness, existing program design, and public acceptability and feasibility (from an organizational and economic perspective) must be considered. In addition, the program must also consider the balance between choice and informed decision making and issues not well addressed by the evidence such as how to best implement CRC screening when there is more than one CRC screening test supported by high-quality evidence. An expert panel comprising members from national and international screening programs, primary care physicians, general surgeons, gastroenterologists, pathologists and laboratory medicine professionals, nurse endoscopists, and members of the public was convened to provide guidance on how to incorporate this evidence in light of the other issues listed above. Their level of agreement with the conclusions is reflected in Table 7. The CCC program will use findings from the evidence summary as well as expert panel recommendations to guide its ongoing development. The specific recommendations resulting from this process have been released recently and can be accessed online at https://www.cancercare.on.ca/common/pages/UserFile.aspx?fileId=358486.

## Supplementary Material

The supplementary material includes the GRADE evidence profiles and meta-analyses comparing fecal tests for occult blood with lower bowel endoscopy, the meta-analyses comparing screening with gFOBT or screening with FS versus no screening, and the meta-analyses comparing screening with FIT versus screening with gFOBT.

## Figures and Tables

**Table 1:** Description of the quality of evidence grades according to Grading of Recommendations, Assessment, Development and Evaluations (GRADE) [9].

Grade	Definition
High	We are very confident that the true effect lies close to that of the estimate of the effect
Moderate	We are moderately confident in the effect estimate: the true effect is likely to be close to the estimate of the effect, but there is a possibility that it is substantially different
Low	Our confidence in the effect estimate is limited: the true effect may be substantially different from the estimate of the effect
Very low	We have very little confidence in the effect estimate: the true effect is likely to be substantially different from the estimate of effect

**Table 2:** Summary of included studies by research question.

Research question and references^*∗*^	Systematic reviews	Outcomes	RCTs	Prospective studies	Retrospective studies	Case-control studies
*Primary question: CRC screening tests* ^*∗∗*^						
gFOBT versus no screening [10–20]	1	CRC mortality	4			
		Complications from tests	3			
		All-cause mortality	4			
		CRC incidence	5			

FS versus no screening [6, 10, 21–28]	1	CRC mortality	4			
		Complications from tests	5			
		All-cause mortality	4			
		CRC incidence	4			

Colonoscopy versus no screening [29–51]	2	CRC mortality		2	1	
		Complications from tests		11	4	
		CRC incidence		2		3

mSEPT9 alone [52]		Diagnostic test accuracy outcomes		1		

Fecal M2-PK alone [53, 54]		Diagnostic test accuracy outcomes		1		1

Stool DNA versus gFOBT or FIT [55–58]		Diagnostic test accuracy outcomes		3		1

FIT versus gFOBT [59–65]	1	Complications from tests	1			
		CRC/advanced adenoma detection rate (ITS)	5			
		CRC/advanced adenoma detection rate (PP)	5			
		Participation rate	6			
		Diagnostic test accuracy outcomes, false-positives/total screened	5			

CT colonography versus colonoscopy [66]		Complications from tests	1			
		CRC/advanced adenoma detection rate (ITS)	1			
		CRC/advanced adenoma detection rate (PP)	1			
		Participation rate	1			

Capsule colonoscopy versus colonoscopy [67]		Complications from tests		1		
		Adenoma detection rate (PP)		1		

Fecal-based tests versus endoscopy [10, 59, 63, 68–80]						
FIT versus colonoscopy	3	Complications from tests	2			
		CRC/advanced adenoma detection rate (ITS)	3			
		Participation rate	3			
FIT versus FS		Complications from tests	1			
		CRC/advanced adenoma detection rate (ITS)	3			
		Participation rate	3			
gFOBT versus colonoscopy		Complications from tests	1			
		Participation rate	2			
gFOBT versus FS		Complications from tests	1			
		CRC/advanced adenoma detection rate (ITS)	2			
		Participation rate	4			
gFOBT versus gFOBT + FS		Complications from tests	1			
		CRC/advanced adenoma detection rate (ITS)	2			
		Participation rate	3			

*Secondary question number 1: age of initiation and cessation by test*						
gFOBT versus no screening [19, 20]		CRC mortality by age group	2			

FS versus no screening [6, 21, 23, 26]		CRC mortality by age group	2			
		CRC incidence by age group	4			

Colonoscopy versus no screening [50]		CRC risk by age group				1

*Secondary question number 2: screening interval by test*						
gFOBT [15, 16, 20]		CRC mortality by interval (annual versus biennial)	1			
		All-cause mortality by interval (annual versus biennial)	1			
		CRC incidence by interval (annual versus biennial)	1			

FIT [81]		CRC/advanced adenoma detection rate by interval (1 versus 2 versus 3 years)	1			
		Participation rate by interval (1 versus 2 versus 3 years)	1			

CRC: colorectal cancer; CT: computed tomographic; DNA: deoxyribonucleic acid; FIT: fecal immunochemical test; FS: flexible sigmoidoscopy; gFOBT: guaiac fecal occult blood test; ITS: intention to screen; PP: per protocol; RCT: randomized controlled trial.

^*∗*^Some RCTs published multiple articles.

^*∗∗*^Tests that were included in our search strategy but yielded no results were not included.

**Table 3:** GRADE evidence profile—gFOBT versus no screening.

Quality assessment							Number of patients		Effect		Quality^1^	Importance
Number of studies	Design	Risk of bias	Inconsistency	Indirectness	Imprecision	Other considerations	gFOBT (cases/person-years)	No screening (cases/person-years)	Relative (95% CI)	Absolute (cases/person-years)		
CRC mortality (followup: range 17–30 years)												
4	Randomised trials	Not serious	Not serious	Not serious	Not serious	Not serious	2027/2674854 (0.08%)	2326/2669246 (0.09%)	**RR 0.87** (0.82 to 0.92)	113 fewer per 1000000 (from 70 fewer to 157 fewer)	*⨁⨁⨁⨁* High	Critical
								Control (gFOBT + FS) = 0.06%^*∗*^		78 fewer per 1000000 (from 48 fewer to 108 fewer)		

Complications from tests (from Holme et al. 2013 [10])^2^												
3	Randomized trials	Not serious	Not serious	Not serious	Not serious	Not serious			N/A^3^		*⨁⨁⨁⨁* High	Critical

All-cause mortality (follow-up: range 17–30 years)												
4	Randomized trials	Not serious	Not serious	Not serious	Not serious	Not serious	74,481/2,674,854 (2.8%)	74,174/2,669,246 (2.8%)	**RR 1** (0.99 to 1.01)	0 fewer per 1,000,000 (from 278 fewer to 278 more)	*⨁⨁⨁⨁* High	Important
								Control (gFOBT + FS) = 1.85%^*∗*^		0 fewer per 1000000 (from 185 fewer to 185 more)		

CRC incidence (follow-up: range 17–30 years)												
5	Randomized trials	Not serious	Not serious	Serious^4^	Not serious	Not serious	4324/2,434,487 (0.2%)	4489/2,431,961 (0.2%)	**RR 0.96 **(0.9 to 1.02)	74 fewer per 1,000,000 (from 37 more to 185 fewer)	*⨁⨁⨁*◯ Moderate	Important
								Control (gFOBT + FS) = 0.16%^*∗*^		64 fewer per 1,000,000 (from 32 more to 160 fewer)		

CI: confidence interval; CRC: colorectal cancer; FS: flexible sigmoidoscopy; gFOBT: guaiac fecal occult blood test; GRADE: Grading of Recommendations, Assessment, Development and Evaluations; ITS: intention to screen; N/A: not applicable; RR: relative risk.

^*∗*^In order to have comparable control rates across all gFOBT and FS trials, the control rates for the no screening groups in the gFOBT and FS trials were combined and calculated from the total number of cases across all gFOBT and FS trials over the total number of person-years across all gFOBT and FS trials.

^1^GRADE working group grades of evidence:

(i) High quality: we are very confident that the true effect lies close to that of the estimate of effect.

(ii) Moderate quality: we are moderately confident in the effect estimate: the true effect is likely to be close to the estimate of the effect, but there is a possibility that it is substantially different.

(iii) Low quality: our confidence in the effect estimate is limited: the true effect may be substantially different from the estimate of the effect.

(iv) Very low quality: we have very little confidence in the effect estimate: The true effect is likely to be substantially different from the estimate of effect.

^2^Major complication defined as bleeding, perforation, or death within 30 days of screening, follow-up colonoscopy, or surgery.

^3^Holme et al. 2013 [10] reported a major complication rate of 0.03%.

^4^Goteborg trial used sigmoidoscopy and double-contrast barium enema as reference standard; other trials used colonoscopy.

**Table 4:** GRADE evidence profile—gFOBT versus FIT.

Quality assessment							Number of patients		Effect		Quality	Importance
Number of studies	Design	Risk of bias	Inconsistency	Indirectness	Imprecision	Other considerations	FIT	gFOBT	Relative (95% CI)	Absolute		
Complications from tests												
1	Randomized trial	Not serious	Not serious	Not serious	Serious^1^	Not serious			Not pooled		*⨁⨁⨁*◯ Moderate	Critical

CRC/advanced adenoma detection rate (ITS)												
5	Randomized trials	Not serious	Not serious	Serious^2^	Not serious	Not serious	278/24,288 (1.1%)	129/27,346 (0.5%)	**RR 2.15** (1.58–2.94)	5 more per 1000 (from 3 more to 9 more)	*⨁⨁⨁*◯ Moderate	Important

CRC/advanced adenoma detection rate (PP)												
5	Randomized trials	Not serious	Not serious	Serious^2^	Not serious	Not serious	278/12,146 (2.3%)	129/10,976 (1.2%)	**RR 1.83** (1.30–2.57)	10 more per 1000 (from 4 more to 18 more)	*⨁⨁⨁*◯ Moderate	Important

False-positive screening test results												
5	Randomized trials	Not serious	Serious^3^	Not serious	Not serious	Not serious	385/24,288 (1.6%)	188/27,346 (0.7%)	**RR 2.12** (1.02–4.39)	8 more per 1000 (from 0 fewer to 23 more)	*⨁⨁⨁*◯ Moderate	Important

Participation rate												
6	Randomized trials	Not serious	Serious^4^	Not serious	Not serious	Not serious	12,271/24,490 (50.1%)	11075/27,548 (40.2%)	**RR 1.16** (1.05–1.28)	64 more per 1000 (from 20 more to 113 more)	*⨁⨁⨁*◯ Moderate	Important

CI: confidence interval; CRC: colorectal cancer; FIT: fecal immunochemical test; gFOBT: guaiac fecal occult blood test; GRADE: Grading of Recommendations, Assessment, Development and Evaluations; ITS: intention to screen; PP: per protocol; RR: relative risk.

^1^Only one study.

^2^Surrogate outcome for CRC mortality.

^3^Heterogeneity: Tau^2^ = 0.61; Chi^2^ = 56.96, df = 3 (*p* < 0.00001); *I* ^2^ = 93%.

^4^Heterogeneity: Tau^2^ = 0.01; Chi^2^ = 107.18, df = 5 (*p* < 0.00001); *I* ^2^ = 95%.

**Table 5:** GRADE evidence profile—FS versus no screening.

Quality assessment							Number of patients		Effect		Quality	Importance
Number of studies	Design	Risk of bias	Inconsistency	Indirectness	Imprecision	Other considerations	FS (cases/person-years)	No screening (cases/person-years)	Relative (95% CI)	Absolute (cases/person-years)		
CRC mortality (follow-up: 6–12 years)												
4	Randomized trials	Not serious	Not serious	Not serious	Not serious	Not serious	576/1,902,184 (0.03%)	1321/3,114,546 (0.04%)	**RR 0.72** (0.65–0.80)	119 fewer per 1,000,000 (from 85 fewer to 148 fewer)	*⨁⨁⨁⨁* High	Critical
								Control (gFOBT + FS) = 0.06%^*∗*^		168 fewer per 1,000,000 (from 120 fewer to 210 fewer)		

Complications from tests (from Holme et al. 2013 [10])^1^												
5	Randomized trials	Not serious	Not serious	Not serious	Not serious	Not serious		N/A^2^			*⨁⨁⨁⨁* High	Critical

All-cause mortality (follow-up: 6–12 years)												
4	Randomized trials	Not serious	Not serious	Not serious	Not serious	Not serious	19,525/1,902,184 (1.0%)	32,903/3,114,546 (1.1%)	**RR 0.97** (0.96–0.99)	317 fewer per 1,000,000 (from 106 fewer to 423 fewer)	*⨁⨁⨁⨁* High	Important
								Control (gFOBT + FS) = 1.85%^*∗*^		555 fewer per 1,000,000 (from 185 fewer to 740 fewer)		

CRC incidence (follow-up: 6–12 years)												
4	Randomized trials	Not serious	Not serious	Not serious	Not serious	Not serious	2218/1,860,990 (0.1%)	4579/3,067,081 (0.1%)	**RR 0.78** (0.74–0.83)	328 fewer per 1,000,000 (from 254 fewer to 388 fewer)	*⨁⨁⨁⨁* High	Important
								Control (gFOBT + FS) = 0.16%^*∗*^		352 fewer per 1,000,000 (from 272 fewer to 416 fewer)		

CI: confidence interval; CRC: colorectal cancer; FS: flexible sigmoidoscopy; gFOBT: guaiac fecal occult blood test; GRADE: Grading of Recommendations, Assessment, Development and Evaluations; RR: relative risk; N/A: not applicable.

^*∗*^In order to have comparable control rates across all gFOBT and FS trials, the control rates for the no screening groups in the gFOBT and FS trials were combined and calculated from the total number of cases across all gFOBT and FS trials over the total number of person-years across all gFOBT and FS trials.

^1^Major complication rate included bleeding, perforation, or death within 30 days of screening, follow-up colonoscopy, or surgery.

^2^Holme et al. 2013 [10] reported a major complication rate of 0.08%.

**Table 6:** GRADE evidence profile—colonoscopy and CRC-related mortality and incidence.

Quality assessment							Effect	Quality	Importance
Number of studies	Design	Risk of bias	Inconsistency	Indirectness	Imprecision	Other considerations	Relative (95% CI)		
CRC mortality (from Brenner et al. 2014) [29]									
3	Observational studies	Serious^1^	Not serious	Not serious	Not serious	Not serious	RR 0.32 (0.23–0.43)	*⨁*◯◯◯ Very low	Critical

Complications from tests (perforations, bleeding, and deaths)									
15	Observational studies	Not serious	Not serious	Not serious	Not serious	Not serious	N/A^2^	*⨁⨁*◯◯ Low	Critical

CRC incidence (from Brenner et al. 2014) [29]									
5	Observational studies	Serious^1^	Serious^3^	Not serious	Not serious	Not serious	RR 0.31 (0.12 to 0.77)	*⨁*◯◯◯ Very low	Important

CI: confidence interval; CRC: colorectal cancer; GRADE: Grading of Recommendations, Assessment, Development and Evaluations; N/A: not applicable.

^1^Mixed study designs included case-control and retrospective.

^2^The risks of perforation or bleeding were less than 1% ranging from 0% to 0.22% for perforations and 0% to 0.19% for bleeding.

^3^Heterogeneity: Tau^2^ = 1.0; (*p* < 0.0001); *I* ^2^ = 94%.

**Table 7:** Responses of the expert panel to the working group's conclusions.

	Reviewer ratings (*N* = 27)				
Conclusions	Strongly disagree (%)	Disagree (%)	Neither agree nor disagree (%)	Agree (%)	Strongly agree (%)
(1) Strong evidence to support use of fecal tests for occult blood to screen people at average risk for CRC	0	0	1 (4)	10 (37)	16 (59)
(2) Strong evidence to support the use of FS to screen people at average risk for CRC	0	0	0	7 (26)	20 (74)
(3) No direct evidence to support the use of colonoscopy to screen people at average risk for CRC, but evidence from FS informs the assessment of benefits and harms of colonoscopy to screen people at average risk for CRC	0	2 (8)	2 (8)	14 (54)	8 (31)
(4) Insufficient evidence to determine how fecal tests for occult blood perform compared with lower bowel endoscopy to screen people at average risk for CRC	0	2 (8)	4 (15)	13 (50)	7 (27)
(5) Insufficient evidence to determine how CT colonography performs compared with colonoscopy to screen people at average risk for CRC	0	0	1 (4)	8 (33)	15 (63)
(6) Insufficient evidence to determine how capsule endoscopy performs compared with colonoscopy to screen people at average risk for CRC	0	0	0	1 (4)	23 (96)
(7) No evidence to support the use of double-contrast barium enema to screen people at average risk for CRC	0	0	2 (8)	0	23 (92)
(8) Insufficient evidence to determine how fecal DNA performs compared with guaiac fecal occult blood test (gFOBT) or fecal immunochemical test (FIT) to screen people at average risk for CRC	0	0	0	8 (33)	16 (67)
(9) Insufficient evidence to support the use of mSEPT9 to screen people at average risk for CRC	0	0	0	2 (8)	23 (92)
(10) Insufficient evidence to support the use of fecal M2-PK to screen people at average risk for CRC	0	0	0	3 (12)	23 (89)
(11) Insufficient evidence to support the use of other metabolomic tests to screen people at average risk for CRC	0	0	1 (4)	2 (9)	20 (87)
(12) Insufficient evidence to support changing ages of initiation and cessation for CRC screening with gFOBT in Ontario	0	1 (4)	1 (4)	9 (36)	14 (56)
(13) Insufficient evidence to recommend an age of initiation or cessation to screen with FS in people at average risk for CRC	0	0	3 (12)	9 (36)	13 (52)
(14) Insufficient evidence to recommend an age of initiation or cessation to screen with colonoscopy in people at average risk for CRC	0	0	4 (16)	10 (40)	11 (44)
(15) Evidence suggests annual or biennial screening using gFOBT in people at average risk for CRC reduces CRC mortality	0	0	1 (4)	11 (42)	14 (54)
(16) Insufficient evidence to recommend an interval to screen people at average risk for CRC using FIT	0	1 (4)	4 (15)	13 (50)	8 (31)
