# Supplementary material for: Colorectal Cancer Screening in Average Risk Populations: Evidence Summary
Source: Can J Gastroenterol Hepatol. 2016 Aug 14;2016:2878149. doi: 10.1155/2016/2878149 (PMC5002289; doi:10.1155/2016/2878149)

**Supplementary Tables and Figures**

**Supplementary Table 1: GRADE evidence profile – one-time FIT versus colonoscopy.**

| **Quality assessment** | | | | | | | **# of patients** | | **Effect** | | **Quality** | **Importance** |
| --- | --- | --- | --- | --- | --- | --- | --- | --- | --- | --- | --- | --- |
|  |  |  |  |  |  |  |  |  |  |  |  |  |
| **# of studies** | **Design** | **Risk of bias** | **Inconsistency** | **Indirectness** | **Imprecision** | **Other considerations** | **FIT** | **Colonoscopy** | **Relative (95% CI)** | **Absolute** |  |  |
| Complications with tests | | | | | | | | | | | | |
| 2 | Randomized trials | Not serious | Serious^1^ | Serious^2^ | Not serious | Not serious |  |  | Not pooled |  | ⨁⨁◯◯ LOW | Critical |
| CRC/advanced adenoma detection rate (ITS) | | | | | | | | | | | | |
| 3 | Randomized trials | Not serious | Serious^3^ | Serious^4^ | Not serious | Not serious | 288/32,908 (0.9%) | 662/32,938 (2.0%) | **RR 0.30** (0.14 to 0.67) | 14 fewer per 1000 (from 7 fewer to 17 fewer) | ⨁⨁◯◯ LOW | Important |
| Participation rate | | | | | | | | | | | | |
| 3 | Randomized trials | Not serious | Serious^5^ | Serious^2^ | Not serious | Not serious | 11,012/32,908 (33.5%) | 6588/32,938 (20.0%) | **RR 1.50** (1.08 to 2.10) | 100 more per 1000 (from 16 more to 220 more) | ⨁⨁◯◯ LOW | Important |

**Abbreviations:** CI = confidence interval; CRC = colorectal cancer; FIT = fecal immunochemical test; GRADE = Grading of Recommendations, Assessment, Development and Evaluations; ITS = intention to screen; RR = relative risk

^1^Different results across studies

^2^Compared only one-time FIT

^3^Heterogeneity: Tau^2^ = 0.34; Chi^2^ = 14.28, df = 2 (p=0.0008); I^2^ = 86%

^4^Compared only one-time FIT; surrogate outcome for CRC mortality

^5^Heterogeneity: Tau^2^ = 0.08; Chi^2^ = 154.54, df = 2 (p<0.00001); I^2^ = 99%

**Supplementary Table 2: GRADE evidence profile – one-time FIT versus FS.**

| **Quality assessment** | | | | | | | **# of patients** | | **Effect** | | **Quality** | **Importance** |
| --- | --- | --- | --- | --- | --- | --- | --- | --- | --- | --- | --- | --- |
|  |  |  |  |  |  |  |  |  |  |  |  |  |
| **# of studies** | **Design** | **Risk of bias** | **Inconsistency** | **Indirectness** | **Imprecision** | **Other considerations** | **FIT** | **FS** | **Relative (95% CI)** | **Absolute** |  |  |
| Complications from tests | | | | | | | | | | | | |
| 1 | Randomized trials | Not serious | Not serious | Serious^1^ | Serious^2^ | Not serious |  |  | Not pooled |  | ⨁⨁◯◯ LOW | Critical |
| CRC/advanced adenoma detection rate (ITS) | | | | | | | | | | | | |
| 3 | Randomized trials | Not serious | Serious^3^ | Serious^4^ | Not serious | Not serious | 139/19077 (0.7%) | 438/25235 (1.7%) | **RR 0.37** (0.21 to 0.67) | 11 fewer per 1000 (from 6 fewer to 14 fewer) | ⨁⨁◯◯ LOW | Important |
| Participation rate | | | | | | | | | | | | |
| 3 | Randomized trials | Not serious | Serious^5^ | Serious^1^ | Not serious | Not serious | 7280/19077 (38.2%) | 7541/25245 (29.9%) | **RR 1.25** (0.82 to 1.89) | 75 more per 1000 (from 54 fewer to 266 more) | ⨁⨁◯◯ LOW | Important |

**Abbreviations:** CI = confidence interval; CRC = colorectal cancer; FIT = fecal immunochemical test; FS = flexible sigmoidoscopy; GRADE = Grading of Recommendations, Assessment, Development and Evaluations; ITS = intention to screen; RR = relative risk

^1^Compared only one-time FIT

^2^Only 1 study

^3^Heterogeneity: Tau^2^ = 0.24; Chi^2^ = 16.87, df = 2 (p=0.0002); I^2^ = 88%

^4^Compared only one-time FIT; surrogate outcome for CRC mortality

^5^Heterogeneity: Tau^2^ = 0.13; Chi^2^ = 463.75, df = 2 (p<0.00001); I^2^ = 100%

**Supplementary Table 3: GRADE evidence profile – one-time gFOBT versus colonoscopy**.

| **Quality assessment** | | | | | | | **# of patients** | | **Effect** | | **Quality** | **Importance** |
| --- | --- | --- | --- | --- | --- | --- | --- | --- | --- | --- | --- | --- |
|  |  |  |  |  |  |  |  |  |  |  |  |  |
| **# of studies** | **Design** | **Risk of bias** | **Inconsistency** | **Indirectness** | **Imprecision** | **Other considerations** | **gFOBT** | **Colonoscopy** | **Relative (95% CI)** | **Absolute** |  |  |
| Complications from tests | | | | | | | | | | | | |
| 1 | Randomized trial | Not serious | Not serious | Serious^1^ | Serious^2^ | Not serious |  |  | Not pooled |  | ⨁⨁◯◯ LOW | Critical |
| Participation rate | | | | | | | | | | | | |
| 2 | Randomized trials | Not serious | Serious^3^ | Serious^1^ | Not serious | Not serious | 1871/6010 (31.1%) | 1930/5894 (32.7%) | **RR 1.13** (0.18 to 6.96) | 43 more per 1000 (from 269 fewer to 1952 more) | ⨁⨁◯◯ LOW | Important |

**Abbreviations:** CI = confidence interval; gFOBT = guaiac fecal occult blood test; GRADE = Grading of Recommendations, Assessment, Development and Evaluations; RR = relative risk

^1^Compared only one-time gFOBT

^2^Only one study

^3^Heterogeneity: Tau^2^ = 1.71; Chi^2^ = 922.55, df = 1 (p<0.00001); I^2^ = 100%

**Supplementary Table 4: GRADE evidence profile – one-time gFOBT versus FS.**

| **Quality assessment** | | | | | | | **# of patients** | | **Effect** | | **Quality** | **Importance** |
| --- | --- | --- | --- | --- | --- | --- | --- | --- | --- | --- | --- | --- |
|  |  |  |  |  |  |  |  |  |  |  |  |  |
| **# of studies** | **Design** | **Risk of bias** | **Inconsistency** | **Indirectness** | **Imprecision** | **Other considerations** | **gFOBT** | **FS** | **Relative (95% CI)** | **Absolute** |  |  |
| Complications from tests | | | | | | | | | | | | |
| 1 | Randomized trials | Not serious | Not serious | Serious^1^ | Serious^2^ | Not serious |  |  | Not pooled |  | ⨁⨁◯◯ LOW | Critical |
| CRC/Advanced adenoma detection rate (ITS) | | | | | | | | | | | | |
| 2 | Randomized trials | Not serious | Not serious | Serious^3^ | Serious^4^ | Not serious | 30/6247 (0.5%) | 114/6238 (1.8%) | **RR 0.29** (0.14 to 0.59) | 13 fewer per 1000 (from 7 fewer to 16 fewer) | ⨁⨁◯◯ LOW | Important |
| Participation rate | | | | | | | | | | | | |
| 4 | Randomized trials | Not serious | Serious^5^ | Serious^1^ | Not serious | Not serious | 4910/10,675 (34.2%) | 2740/8558 (31.9%) | **RR 1.31** (0.91 to 1.89) | 99 more per 1000 (from 29 fewer to 285 more) | ⨁⨁◯◯ LOW | Important |

**Abbreviations:** CI = confidence interval; CRC = colorectal cancer; FS = flexible sigmoidoscopy; gFOBT = guaiac fecal occult blood test; GRADE = Grading of Recommendations, Assessment, Development and Evaluations; ITS = intention to screen; RR = relative risk

^1^Compared only one-time gFOBT

^2^Only one study

^3^Compared only one-time gFOBT; surrogate outcome for CRC mortality

^4^Few events

^5^Heterogeneity: Tau^2^ = 0.14; Chi^2^ = 238.42, df = 3 (p<0.00001); I^2^ = 99%

**Supplementary Table 5: GRADE evidence profile – one-time gFOBT versus gFOBT+FS**.

| **Quality assessment** | | | | | | | **# of patients** | | **Effect** | | **Quality** | **Importance** |
| --- | --- | --- | --- | --- | --- | --- | --- | --- | --- | --- | --- | --- |
|  |  |  |  |  |  |  |  |  |  |  |  |  |
| **# of studies** | **Design** | **Risk of bias** | **Inconsistency** | **Indirectness** | **Imprecision** | **Other considerations** | **gFOBT** | **gFOBT+FS** | **Relative (95% CI)** | **Absolute** |  |  |
| Complications from tests | | | | | | | | | | | | |
| 1 | Randomized trials | Not serious | Not serious | Serious^1^ | Serious^2^ | Not serious |  |  | Not pooled |  | ⨁⨁◯◯ LOW | Critical |
| CRC/advanced adenoma detection rate (ITS) | | | | | | | | | | | | |
| 2 | Randomized trials | Not serious | Not serious | Serious^3^ | Serious^4^ | Not serious | 24/8611 (0.3%) | 113/8738 (1.3%) | **RR 0.21** (0.14 to 0.33) | 10 fewer per 1000 (from 9 fewer to 11 fewer) | ⨁⨁◯◯ LOW | Important |
| Participation rate | | | | | | | | | | | | |
| 3 | Randomized trials | Not serious | Serious^5^ | Serious^1^ | Not serious | Not serious | 5012/9856 (50.9)% | 3247/9988 (32.5)% | **RR 1.54** (0.98 to 2.40) | 176 more per 1000 (from 7 fewer to 455 more) | ⨁⨁◯◯ LOW | Important |

**Abbreviations:** CI = confidence interval; CRC = colorectal cancer; FS = flexible sigmoidoscopy; gFOBT = guaiac fecal occult blood test; GRADE = Grading of Recommendations, Assessment, Development and Evaluations; ITS = intention to screen; RR = relative risk

^1^Compared only one-time gFOBT

^2^Only 1 study

^3^Compared only one-time gFOBT; surrogate outcome for CRC mortality

^4^Few events

^5^Heterogeneity: Tau^2^ = 0.15; Chi^2^ = 223.97, df = 2 (p<0.00001); I^2^ = 99%

Supplementary Figure 1: Meta-analysis of guaiac fecal occult blood test (gFOBT) versus no screening: Colorectal cancer mortality.


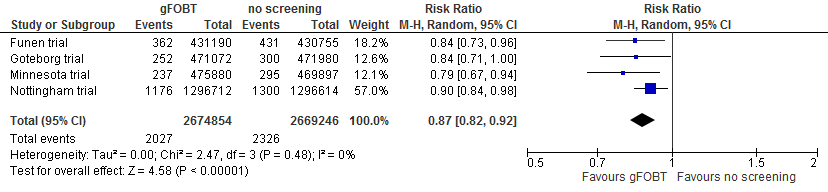


Supplementary Figure 2: Meta-analysis of gFOBT versus no screening: All-cause mortality.


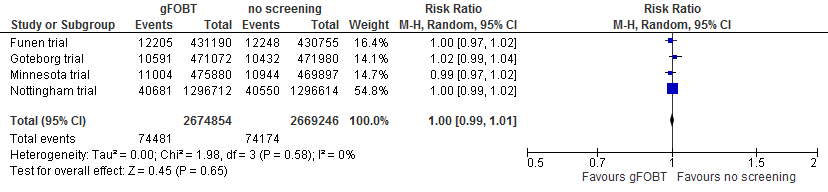


Supplementary Figure 3: Meta-analysis of gFOBT versus no screening: Colorectal cancer incidence.


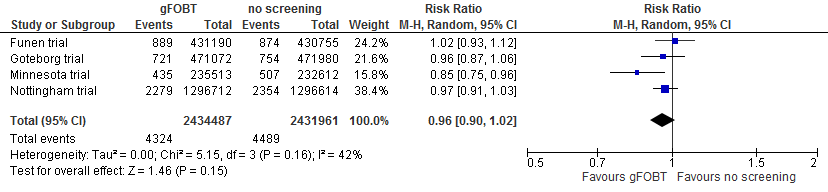


**Supplementary Figure 4: Meta-analysis of** **flexible sigmoidoscopy (FS) versus no screening: Colorectal cancer mortality.**

**
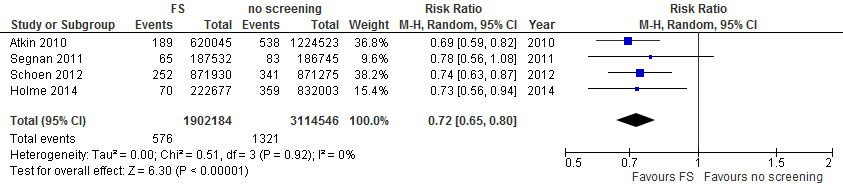
**

**Supplementary Figure 5: Meta-analysis of FS versus no screening (Schoen 2012 excludes death due to prostate, lung and ovarian cancer): All-cause mortality.**
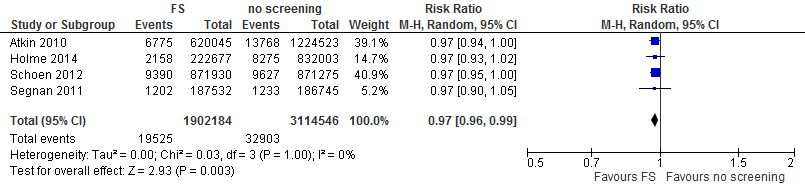


**Supplementary Figure 6: Meta-analysis of** **FS versus no screening: Colorectal cancer incidence.**


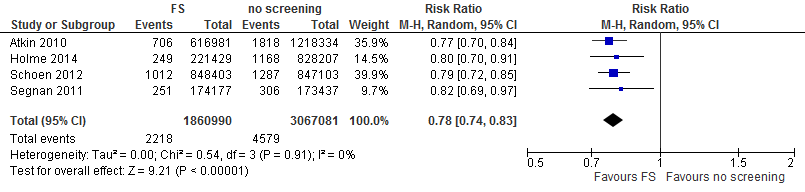


**Supplementary Figure 7: Meta-analysis of** **fecal immunochemical test** **(FIT) versus gFOBT: Colorectal cancer/advanced adenoma detection rate (intention to screen).**


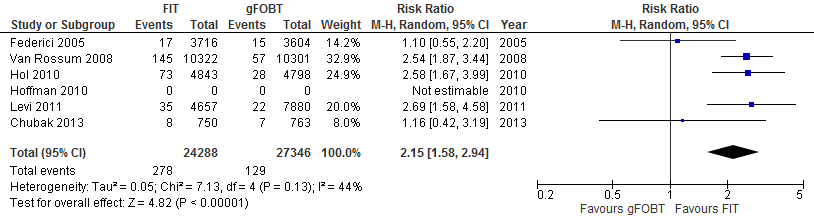


**Supplementary Figure 8: Meta-analysis of** **FIT versus gFOBT: Colorectal cancer/advanced adenoma detection rate (per protocol).**

**
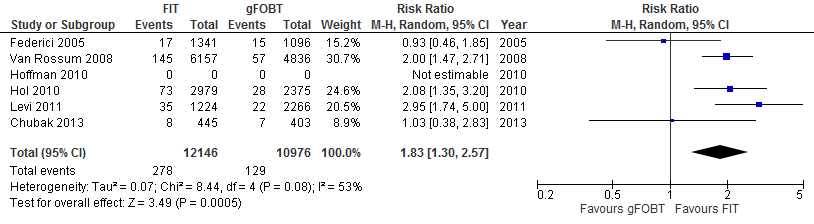
**

**Supplementary Figure 9: Meta-analysis of** **FIT versus gFOBT: False-positive/total screened test results.
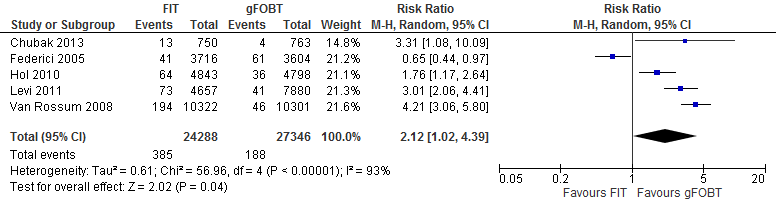
**

**Supplementary Figure 10: Meta-analysis of FIT versus gFOBT: Participation rate.**

**
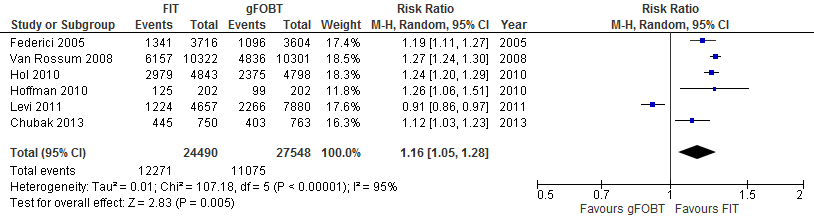
**

**Supplementary Figure 11. Meta-analysis of** **FIT versus colonoscopy: Advanced neoplasia (intention to screen).**


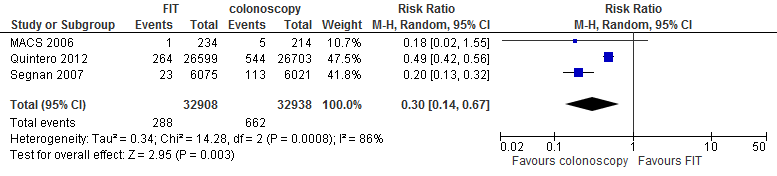


**Supplementary Figure 12. Meta-analysis of** **FIT versus colonoscopy: Participation rate.**


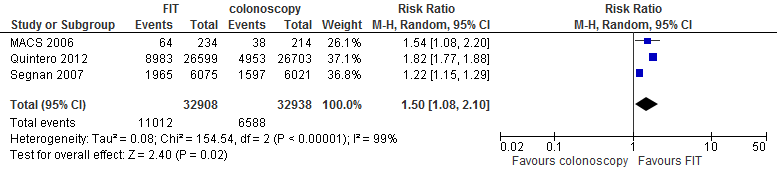


**Supplementary Figure 13. Meta-analysis of** **FIT versus FS: Advanced neoplasia (intention to screen).**
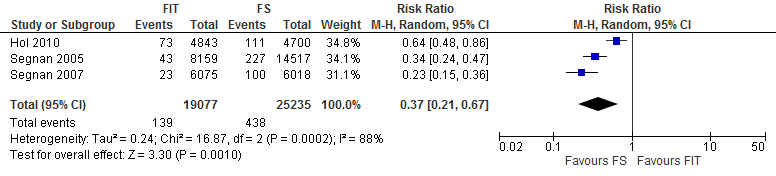


**Supplementary Figure 14. Meta-analysis of** **FIT versus FS: Participation rate.**


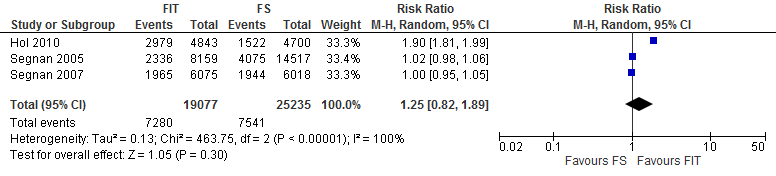


**Supplementary Figure 15. Meta-analysis of gFOBT versus colonoscopy: Participation rate.**
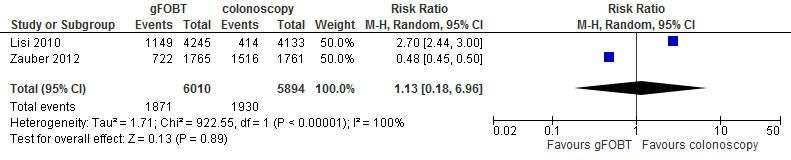


**Supplementary Figure 16. Meta-analysis of** **gFOBT versus FS: Advanced neoplasia (intention to screen).**


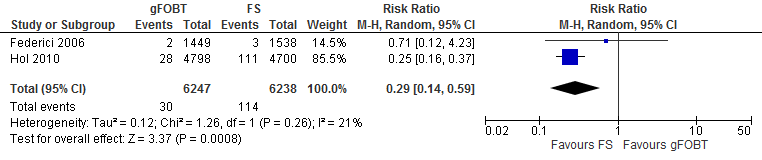


**Supplementary Figure 17. Meta-analysis of** **gFOBT versus FS: Participation rate.**
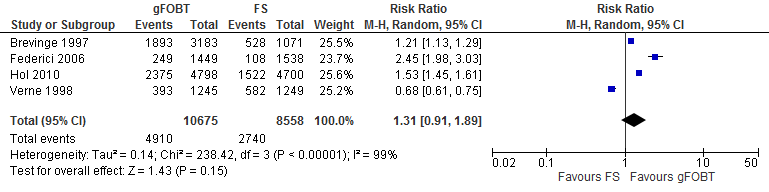


**Supplementary Figure 18. Meta-analysis of** **gFOBT versus gFOBT + FS: Advanced neoplasia (intention to screen).**
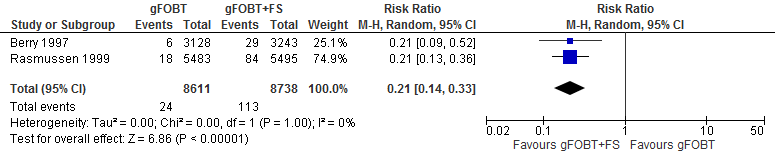


**Supplementary Figure 19. Meta-analysis of** **gFOBT versus gFOBT + FS: Participation rate.**
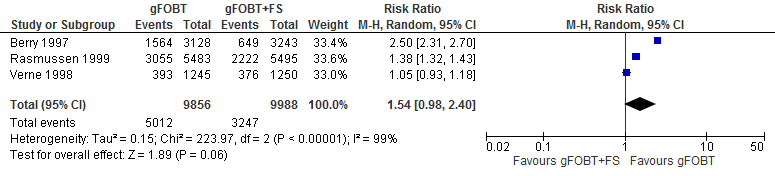

Supplement: Supplementary file 1 — The supplementary material includes the GRADE evidence profiles and meta-analyses comparing fecal tests for occult blood with lower bowel endoscopy, the meta-analyses comparing screening with gFOBT or screening with FS versus no screening, and the meta-analyses comparing screening with FIT versus screening with gFOBT. [file 2878149.f1.docx]
